# Del Nido cardioplegia versus cold blood cardioplegia in adult cardiac surgery: a meta-analysis of randomized clinical trials

**DOI:** 10.1186/s13019-024-02846-0

**Published:** 2024-06-22

**Authors:** Congcong Li, Haiyan Xiang, Heng Yang, Wu Liu, Wanqi Lan, Chao Luo, Shuangjian Han, Yongqin Li, Yanhua Tang

**Affiliations:** Department of Cardiovascular Surgery, The Second Affiliated Hospital, Jiangxi Medical College, Nanchang University, Nanchang, Jiangxi Province 330006 China

**Keywords:** Cardioplegia, Myocardial protection, Randomized controlled trials, Meta-analysis

## Abstract

**Objective:**

Systematic evaluation of the safety of del Nido cardioplegia compared to cold blood cardioplegia in adult cardiac surgery.

**Methods:**

We systematically searched PubMed, EMbase, The Cochrane Library and ClinicalTrials.gov for randomized clinical trials (published by 14 January 2024) comparing del Nido cardioplegia to cold blood cardioplegia in adult. Our main endpoints were myocardial injury markers and clinical outcomes. We assessed pooled data by use of a random-effects model or a fixed-effects model.

**Results:**

A total of 10 studies were identified, incorporating 889 patients who received del Nido cardioplegia and 907 patients who received cold blood cardioplegia. The meta-analysis results showed that compared with the cold blood cardioplegia, the del Nido cardioplegia had less volume of cardioplegia, higher rate of spontaneous rhythm recovery after cross clamp release, lower levels of postoperative cardiac troponin T and creatinine kinase-myocardial band, all of which were statistically significant. However, there was no statistically significant difference in postoperative troponin I and postoperative left ventricular ejection fraction. The clinical outcomes including mechanical ventilation time, intensive care unit stay time, hospital stay time, postoperative stroke, postoperative new-onset atrial fibrillation, postoperative heart failure requiring intra-aortic balloon pump mechanical circulation support, and in-hospital mortality of both are comparable.

**Conclusion:**

Existing evidence suggests that del Nido cardioplegia reduced volume of cardioplegia administration and attempts of defibrillation. The superior postoperative results in CTnT and CK-MB may provide a direction for further research on improvement of the composition of cardioplegia.

**Visual Abstract:**

**Figure:**
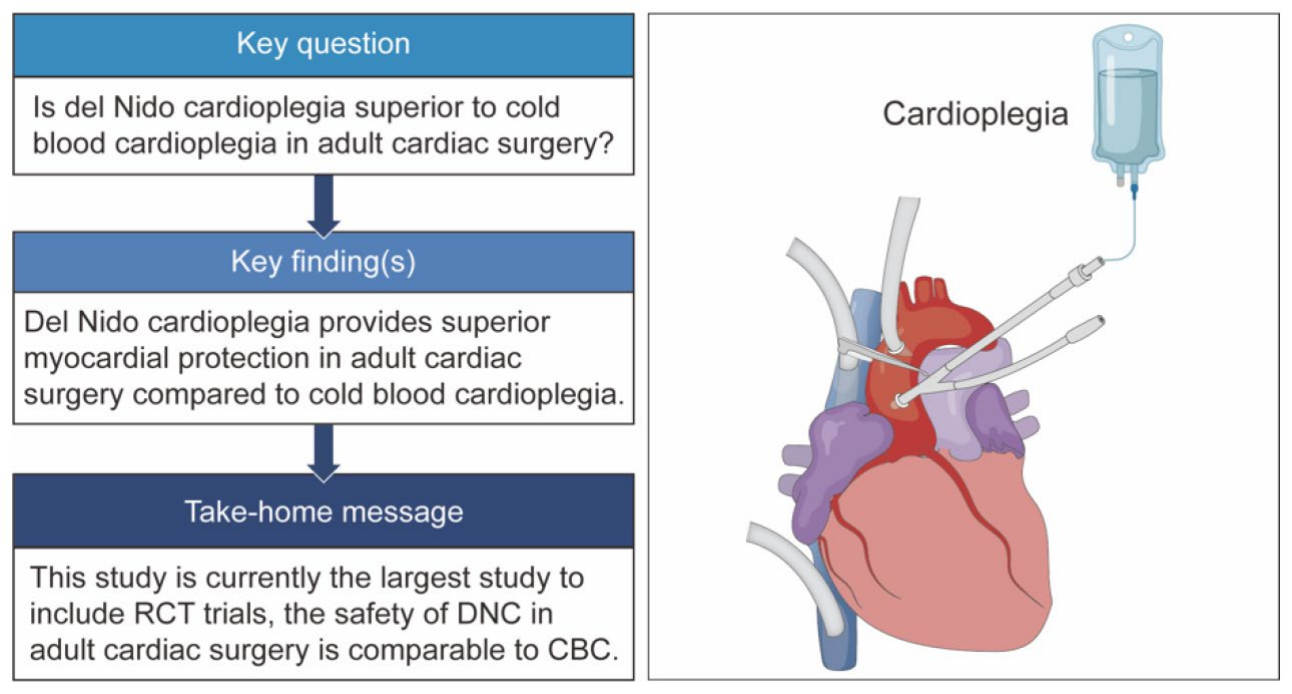

**Supplementary Information:**

The online version contains supplementary material available at 10.1186/s13019-024-02846-0.

## Introduction

Mechanical arrest using cardioplegia remains the current gold standard for cardiac protection during cardiac surgery [1]. Cardioplegia can decrease myocardial energy consumption and ischemia reperfusion injury [2]. However, the selection of cardioplegia is entirely based on the experience and habits of the surgeons or perfusionist, and there is currently no unified guideline as a reference [3]. Myocardial protection has always been a daunting challenge for cardiac surgeons. Del Nido Cardioplegia (DNC) is an extracellular myocardial protection fluid that provides up to 90 min of myocardial protection after a single dose at 20 ml/kg [4]. In the early 1990s, Pedro del Nido and his team developed this solution to protect immature myocardial cells [5]. Delivery of del Nido solution was administered in a 1:4 ratio of blood to crystalloid [6]. In the past few decades, del Nido cardioplegia has been widely used for intraoperative myocardial protection of congenital heart disease [7]. Based on pediatric usage experience, del Nido was quickly applied to adult cardiac surgery for cardiac protection. Compared to traditional cold blood cardioplegia (blood to crystalloid ratio of 4:1), this solution has a higher dilution, the hallmark of del Nido cardioplegia is lidocaine supplement which acts as a sodium channel blocker that reduces intracellular sodium and calcium accumulation [8]. However, there are some physiological characteristic differences in tolerance of calcium damage and ischemia between infants and adults. It has not been universally acknowledged whether del Nido cardioplegia is safe for adults. This study systematically evaluates the safety of del Nido cardioplegia and cold blood cardioplegia in adult patients undergoing cardiac surgery from the perspectives of myocardial protection and clinical outcomes, in order to provide new evidence for the application of del Nido cardiac arrest solution in adult cardiac surgery.

## Materials and methods

### Study design and inclusion/exclusion criteria

This systematic review and meta-analysis were performed in accordance with the Preferred Reporting Items for Systematic Review and Meta-Analysis (PRISMA) Statement and was registered in PROSPERO (ID: CRD42024502507). We applied the PICOS (Population, Intervention, Comparison, Outcome and Study design) criteria to define inclusion criteria:

1. Population: adult patients who underwent cardiac surgery requiring cardiopulmonary bypass (CPB) and myocardial arrest, regardless of race or nationality.
2. Intervention: Cardioplegia (DNC) was used for cardiac arrest.
3. Comparison: cold blood cardioplegia (CBC) was used for cardiac arrest.
4. Outcome: ⑴Volume of cardioplegia (L); ⑵CPB time (minute); ⑶Aortic cross-clamp (ACC) time (minute); ⑷ Ventricular fibrillation after aortic cross-clamp removal; ⑸ Postoperative Cardiac Troponin I (CTnI) at 24 h after surgery (ng/ml); ⑹Cardiac Troponin T (CTnT) at 24 h after surgery (ng/ml); ⑺ Postoperative Creatinine Kinase-Myocardial Band (CK-MB) at 24 h after surgery (U/L); ⑻Postoperative left ventricular ejection fraction (LVEF); ⑼Mechanical ventilation time (hour); ⑽Intensive care unit (ICU) stay time (day); ⑾Hospital stay time (day); ⑿Postoperative new-onset atrial fibrillation; ⒀Postoperative stroke; ⒁ Postoperative intra-aortic balloon pump (IABP) requirement; ⒂In-hospital mortality.
5. Study design: randomized clinical trial.

Exclusion criteria was defined as follows: ⑴Non English literature; ⑵Repeated publication literature; ⑶Literature with incomplete original data.

### Search strategy

A comprehensive literature retrieval was performed by searching PubMed, EMbase, the Cochrane Library, and ClinicalTrials.gov using search terms “del Nido” or “del-nido” and “cardioplegia” or “cardioplegic solution”. We selected randomized clinical trial (RCT) studies on the safety of del Nido cardioplegia compared to cold blood cardioplegia in adult cardiac surgery, the search deadline is from database establishment to 14 January 2024. The complete search used for PubMed was: ((((del Nido) OR (del-nido)) AND (cardioplegia)) OR (cardioplegic solution)) OR (cardioplegia solution). We considered all potentially eligible researches for review, regardless of the primary outcome or language. We also did a manual search, using the reference lists of key articles published in English.

### Data extraction and tabulation

Two double-blinded researchers (Congcong Li and Haiyan Xiang) independently screened literature, extracted data, tabulated intervention characteristics, and cross checked. If there are differences, they can be resolved through discussion or negotiation with third parties (Yanhua Tang). When selecting literature, first read the title, and after excluding obviously unrelated literature, further read the abstract and full text to determine whether to include it. If necessary, contact the original research author via email or phone to obtain uncertain but important information for this study. The content of data extraction includes: ⑴Basic information of the included research: research title, first author, publication journal, publication year, etc.; ⑵Baseline characteristics and intervention of the research subjects; ⑶The key elements of bias risk assessment; ⑷Outcome indicators and outcome measurement data of concern.

### Quality of evidence assessment

Two independent researchers assessed the risk for bias included in the study and cross checked the results. The bias risk assessment for included RCT studies was conducted using the bias risk assessment tool recommended in Cochrane Handbook 5.1.0.

### Statistical analysis

Meta-analysis was performed using RevMan5.3 software. Measurement data were reported as mean ± standard deviation (SD) or median (interquartile range), Comparative data between cohorts are shown as mean differences (MD), and relative risk (RR) was used as effect analysis statistic for dichotomous variables, with 95% confidence interval (CI) provided for each effect size. Heterogeneity among the included studies was analyzed using the χ^2^ (Q) test (with a test level of α = 0.1), and the heterogeneity was evaluated by combining I^2^. When the P value of Q test was less than 0.1 or I^2^ was greater than 50%, significant heterogeneity was shown among the studies, and the random effect model was used for Meta-analysis; otherwise, the fixed effect model was used. If there was statistical heterogeneity among the studies, the source of heterogeneity was further analyzed, and the random effect model was used for analysis after excluding the influence of obvious clinical heterogeneity. *P* < 0.05 was considered statistically significant. Obvious clinical heterogeneity was processed by subgroup analysis or sensitivity analysis, or descriptive analysis only.

## Results

### Study selection and baseline characteristics

**Fig. 1:**
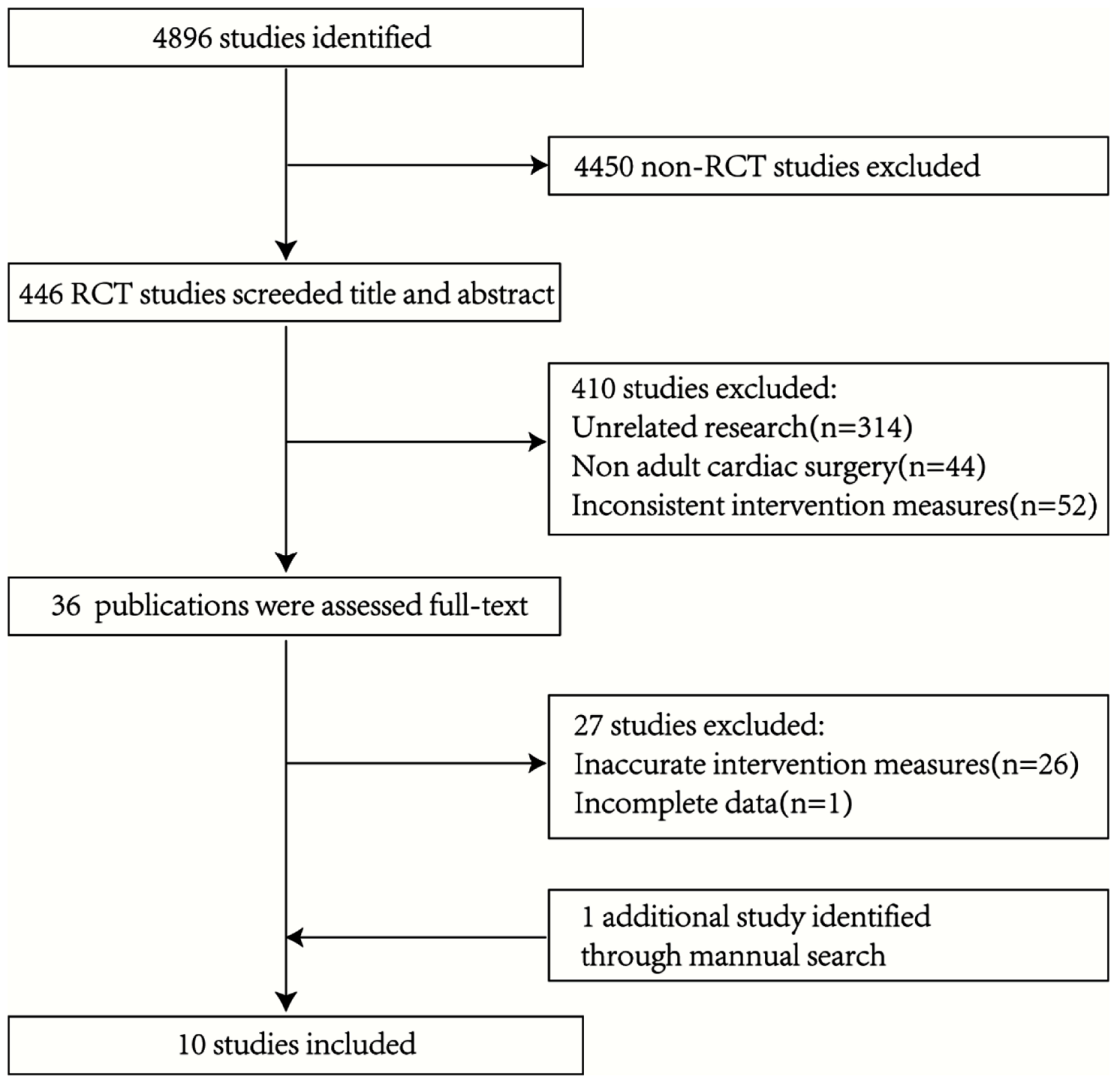
PRISMA flowchart of studies included in the meta-analysis

A total of 4896 relevant literature were obtained in the initial examination, and after layer by layer screening, 10 RCT studies [4, 9–17] were finally included, with a total of 1796 adult patients, including 889 patients in the DNC group and 907 patients in the CBC group. The literature screening process and results are shown in Fig. 1. The basic characteristics of the included studies are shown in Table 1.

**Table 1:** Characteristics of included studies

Author & year	Country	Study period	Simple (*N*)	Gender (Male, *N*)		Mean age (year)		Operation procedure	Hypertension (*N*) DNC/CBC	Diabetes (*N*) DNC/CBC	Clinical outcome
				DNC	CBC	DNC	CBC				
Garcia-Suarez 2022 [16]	Spain	2018.06-2019.09	474	143	148	65.60 ± 11.94	65.30 ± 11.93	Mix	147/151	17/21	⑵⑶⑷⑹⑻⑼⑽⑾⑿⒀⒁⒂
Zhang 2022 [17]	China	2021.01-2021.09	133	37	36	54.50 ± 7.21	56.20 ± 6.71	Mix	19/18	NA	⑴⑵⑶⑷⑸⑺⑻⑼⑽⑿
Demir 2022 [15]	Turkey	2019.07-2021.02	213	79	88	60.56 ± 10.24	61.07 ± 9.36	CABG	61/66	47/38	⑿⒀⒁⒂
Moktan Lama 2021 [13]	Nepal	2018.05-2019.09	90	36	38	59.98 ± 8.96	57.51 ± 9.71	CABG	16/19	15/20	⑴⑵⑶⑸⑺⑻⑼⑽⑾⒂
Urcun 2021 [14]	Turkey	2017.01-2020.01	300	114	126	57.64 ± 12.66	61.78 ± 11.33	CABG	72/60	45/27	⑵⑶⑹⑺⑻⑼⑽⑾⑿⒁⒂
Gunaydin 2020 [12]	USA	2017.01-2019.06	220	61	72	71.00 ± 8.00	73.00 ± 10.00	CABG	83/92	72/80	⑴⑶⑻⑼⑽⑾⑿⒁⒂
Kirisci 2020 [11]	Turkey	2019.07-2020.01	60	20	22	59.03 ± 11.58	62.00 ± 11.53	CABG	14/13	12/14	⑴⑵⑶⑷⑽⑾⑿⒂
Sanetra 2019 [4]	Poland	2016.06-2018.06	150	48	38	62.53 ± 12.69	63.83 ± 10.99	AVR	50/51	13/16	⑴⑵⑶⑷⑹⑺⑻⑾⑿⒀⒂
Kantathut 2019 [10]	Thailand	2017.02-2017.11	89	24	32	64.85 ± 9.96	65.94 ± 9.96	Mix	36/36	17/15	⑴⑵⑶⑷⑹⑺⑻⑽⑿⒀⒁⒂
Ad 2018 [9]	USA	2015.02-2016.04	89	40	31	65.30 ± 7.90	65.10 ± 9.10	CABG/VS	35/32	22/15	⑴⑵⑶⑷⑸⒀

DNC: del Nido cardioplegia, CBC: cold blood cardioplegia, Mix: mix operation, CABG: coronary artery bypass grafting, AVR: atrial valve replacement, VS: valve surgery, ⑴Volume of cardioplegia; ⑵Cardiopulmonary bypass time; ⑶Aortic cross-clamp time; ⑷ Ventricular fibrillation after aortic cross-clamp removal; ⑸ 24 h postoperative Troponin I levels; ⑹ Troponin T levels at 24 h after surgery; ⑺24 h postoperative Creatinine Kinase-Myocardial Band levels; ⑻Postoperative left ventricular ejection fraction; ⑼Mechanical ventilation time; ⑽ Intensive care unit stay time; ⑾Hospital stay time; ⑿Postoperative new-onset atrial fibrillation; ⒀Postoperative stroke; ⒁ postoperative heart failure requiring intra-aortic balloon pump mechanical circulation support; ⒂In-hospital mortality

The 10 RCT studies were all published between 2018 and 2023 (Table 1). Mean trial duration was 18 months (range 9 months to 36 months). More than half of patients underwent Coronary artery bypass grafting (CABG) surgery and three included studies focusing on mixed adult cardiac surgery procedures, one trail focused on aortic valve replacement (AVR) surgery, and one trail included adult patients who underwent CABG or valve surgery (VS) surgery. All 10 randomized trials were single-center, one study was stopped early [9], one study was s finished with a delay due to the coronavirus pandemic situation [15]. 3 were registered in Clinicaltrials.gov [4, 9, 16], and 1 was registered in Thai clinical trial registry [10], 5 trials reported detailed descriptions of methods for generating random sequence [9, 12, 14–16], 3 trials specified blind method for participants [4, 12, 16]. The results of bias risk assessment of included studies are shown in Supplementary Figures S1.

### Intraoperative outcome

**Fig. 2:**
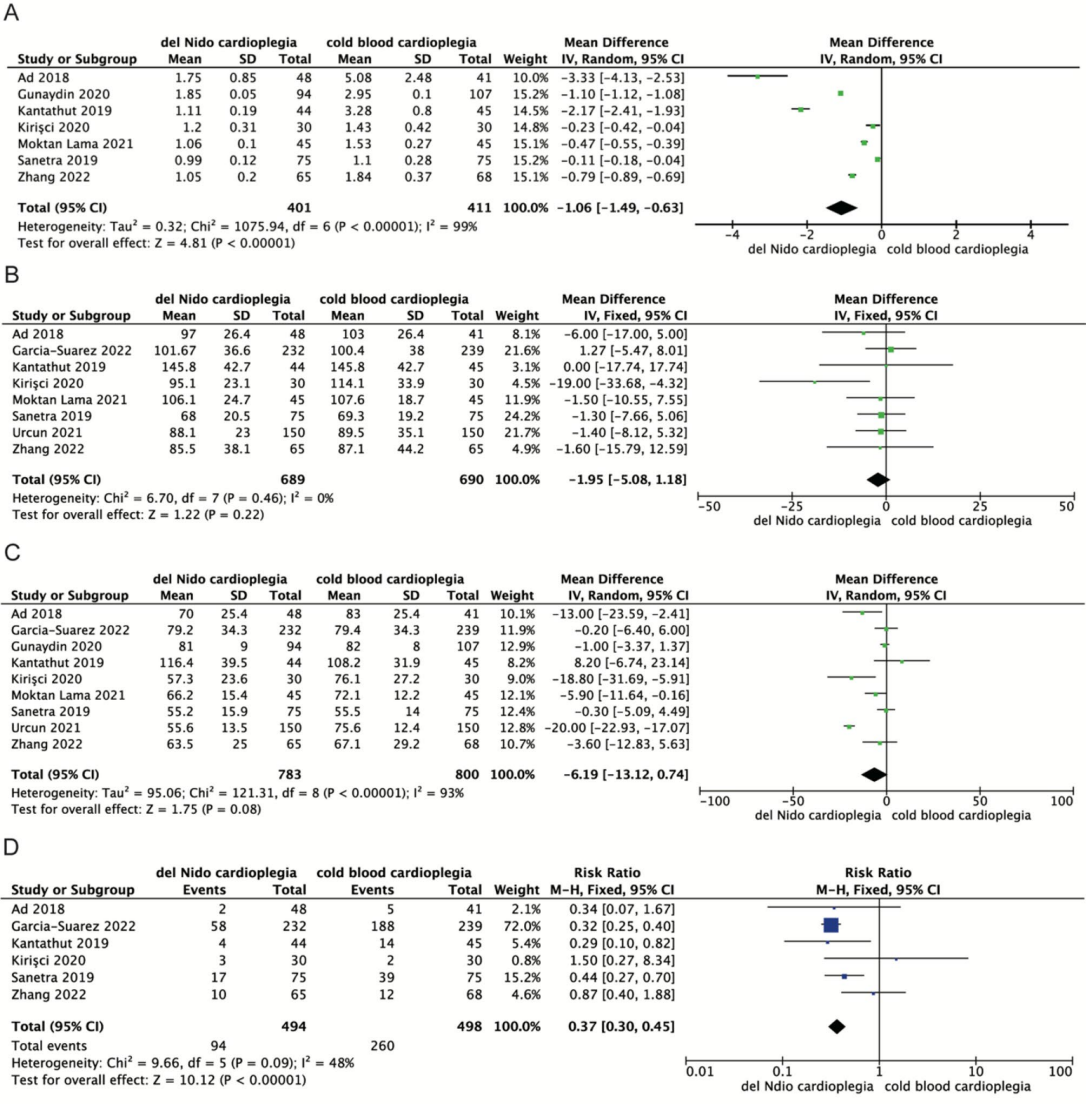
Meta-analyses of del Nido cardioplegia versus cold blood Cardioplegia, comparing the cardioplegia volume, CPB time, ACC time and intraoperative defibrillation. **A**, Cardioplegia volume; **B**, CPB time; **C**, ACC time; **D**, Defibrillation

Seven studies assessed intraoperative reperfusion volume of cardioplegia. Pooling data of these studies showed that DNC led to lower volume during operation than CBC [MD=-1.06, 95%CI (− 1.49, -0.63), *P* < 0.0001] (Fig. 2). Six studies assessed ventricular fibrillation after aortic cross-clamp removal. The results of meta-analysis showed that the defibrillation requirement was lower in the DNC group (Fig. 2). and the DNC group had a higher spontaneous cardiac rhythm recovery rate [MD = 0.37, 95%CI (0.30, 0.45), *P* < 0.00001]. Eight and nine studies respectively assessed CPB time and ACC time. Pooling the data of these studies showed no significant difference in the CPB time [MD=-1.95, 95%CI (-5.08, 1.18), *P* = 0.22] and ACC time [MD=-6.19, 95%CI (-13.12, 0.74), *P* = 0.08] with DNC compared with CBC (Fig. 2).

### Levels of myocardial injury biomarker and postoperative cardiac function

**Fig. 3:**
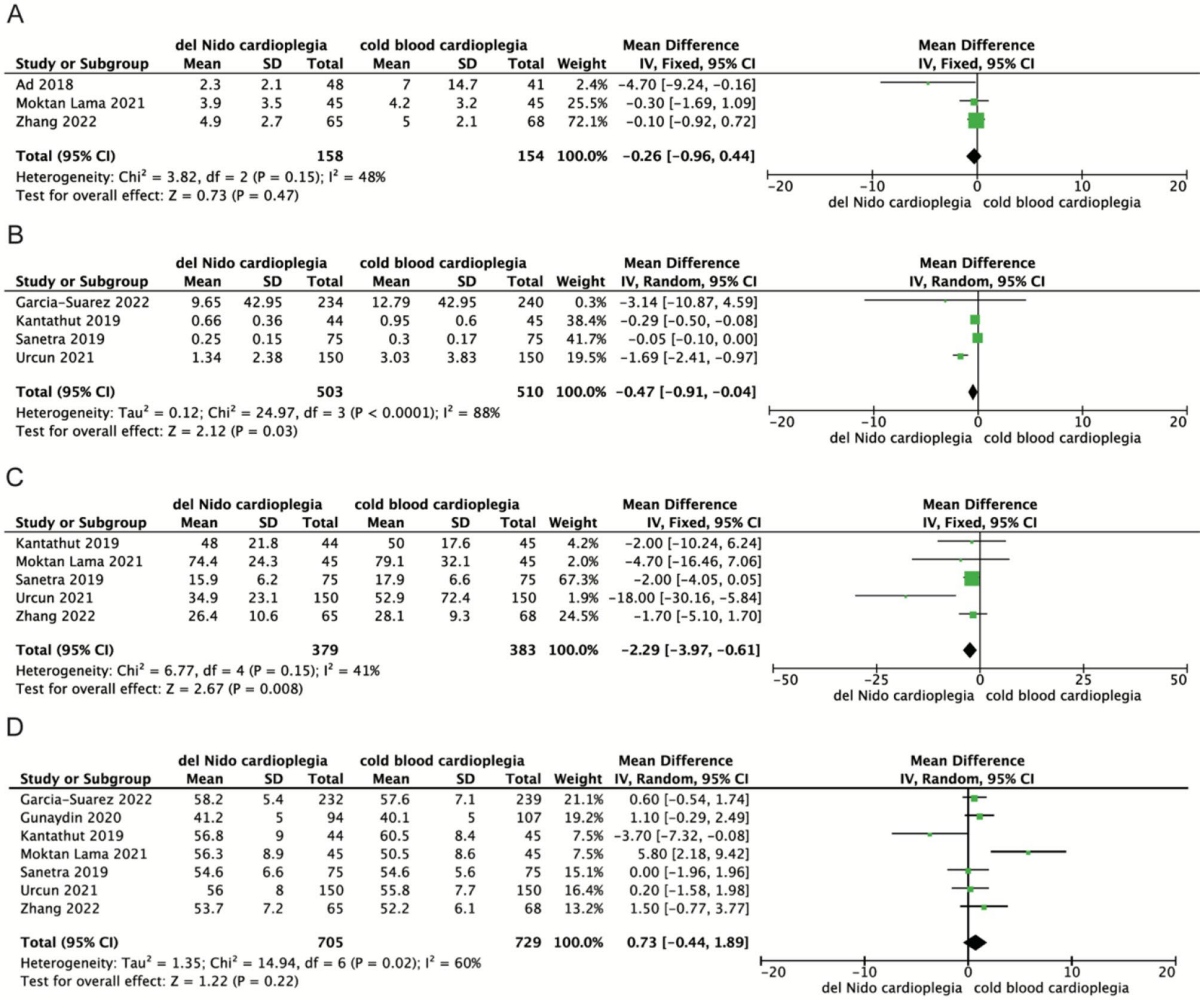
Meta-analyses of del Nido cardioplegia versus cold blood Cardioplegia, comparing the levels of myocardial injury markers 24 h after surgery and postoperative left ventricular ejection fraction. **A**, CTn I; **B**, CTn T; **C**, CK-MB; **D**, LVEF

Three, four and five studies assessed the levels of CTnI, CTnT and CK-MB at 24 h after surgery, respectively. The results showed that compared with the CBC group, the CTnI level in the DNC group had no significant difference [MD= -0.26, 95%CI (-0.96, 0.44), *P* = 0.47]. The CTnT level and CK-MB level in the DNC group were significantly lower than those in the CBC group [MD=-0.47, 95%CI (-0.91, -0.04), *P* = 0.03; MD=-2.29, 95%CI (-3.97, -0.61), *P* = 0.008] (Fig. 3).

Seven studies assessed postoperative LVEF, there were no differences in preoperative ejection fraction in selected studies. Pooling the data of these studies showed that the postoperative LVEF in DNC group was better than that in CBC group, and the difference between the two groups was not statistically significant [MD = 0.73, 95%CI (-0.44, 1.89), *P* = 0.22] (Fig. 3).

### Postoperative clinical outcome

**Fig. 4:**
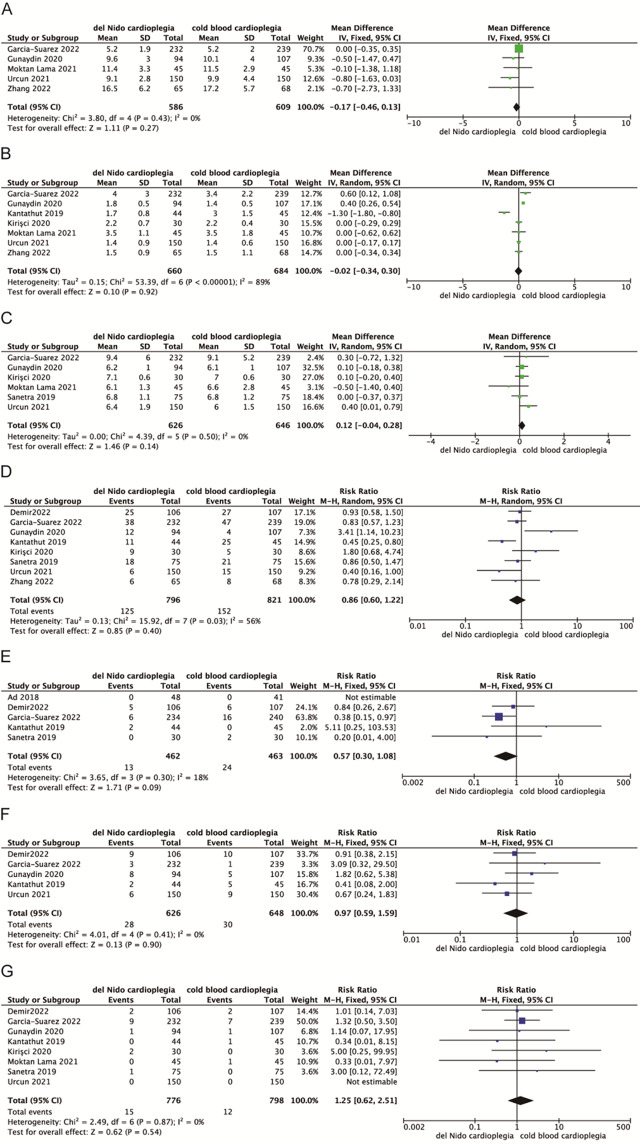
Meta-analyses of del Nido cardioplegia versus cold blood Cardioplegia, comparing clinical outcome. Outcomes assessed are (**A**) Mechanical ventilation time, (**B**) Intensive care unit stay time, (**C**) Hospital stay time, (**D**) Postoperative new-onset atrial fibrillation, (**E**) Postoperative stroke, (**F**) postoperative heart failure requiring intra-aortic balloon pump mechanical circulation support, and (**G**) In-hospital mortality. For each estimate, the grey shaded area is the weight of the estimate in proportion to the overall effect

Five, seven and six studies were included in the evaluation of mechanical ventilation time, ICU stay time and hospital stay, respectively. The results of meta-analysis showed that there were no statistically significant differences in mechanical ventilation time, ICU stay time and hospital stay between DNC group and CBC group [MD=-0.17, 95%CI (-0.46, -0.13), *P* = 0.27; MD=-0.02, 95%CI (-0.34, -0.30), *P* = 0.92; MD = 0.12, 95%CI (-0.04, 0.28), *P* = 0.14] (Fig. 4).

The incidence of postoperative adverse events such as new atrial fibrillation, stroke, IABP support, and hospital death were evaluated. The results of meta-analysis showed that there were no statistically significant differences between the DNC group and the CBC group in postoperative adverse events, including postoperative new-onset atrial fibrillation, stroke, IABP requirement and in-hospital mortality[MD = 0.86, 95%CI (0.60, 1.22), *P* = 0.4; MD = 0.56, 95%CI (0.30, 1.08), *P* = 0.09; MD = 0.97, 95%CI(0.59, 1.59), *P* = 0.90; MD = 1.25, 95%CI (0.62, 2.51), *P* = 0.54] (Fig. 4).

### Sensitivity analysis and publication bias assessment

Sensitivity analysis showed that there was no significant change in the combined effect size of outcome indicators after one article was excluded separately. The funnel-plot was used to evaluate publication bias, the points in the image converge into a roughly symmetrical (inverted) funnel (Supplementary Figures S2).

## Discussion

It is necessary to quickly arrest the heart before cardiac surgery. In order to better restore the function of myocardium after ischemia, the concept of cardioplegia is proposed [18]. With the development of cardiac surgery, the research on myocardial protection has been going on for decades, and the establishment of the overall theoretical system of myocardial protection and the clinical practice methods are becoming more and more mature. The three important components of cardiac arrest fluid to protect myocardium include: (1) Increase the extracellular K^+^ and Mg^2+^ concentrations, and reduce the inflow of Na^+^ and Ca^2+^ to reduce their intracellular concentrations; (2) low temperature to reduce the needs of cell metabolism; (3) Nutrients [8]. The commonly used cardioplegia solution in clinical practice includes cold blood cardioplegia (CBC) prepared with high potassium solution and blood at 1:4, intracellular histidine-tryptophane-ketoglutarate solution, extracellular liquid St.Thomas solution, etc. Del Nido cardioplegia was used in adults as an extracellular cardioplegia has been a new trend, which is formulated with high potassium crystal fluid and blood in a 4:1 formula. It has lower viscosity than traditional cold blood cardioplegia and can provide better perfusion of the myocardium [19]. It was originally designed for the immature myocardium of children, because only a single perfusion can provide myocardial protection for 90 min, ensure the continuity of surgery, and have a clear protective effect on the immature myocardium of children. In recent years, this cardioplegia solution has also been applied in adult cardiac surgery. Several studies have shown that del Nido cardioplegia solution also has high safety and effectiveness in adult cardiac surgery. Del Nido cardioplegia applied in adults is a new method of myocardial protection, while the clinical application of del Nido cardioplegia for mature myocardium protection is still insufficient.

This study was a meta-analysis of RCT studies of del Nido cardioplegia and cold blood cardioplegia in adult cardiac surgery. The result showed that del Nido cardioplegia was correlated with cardioplegia perfusion volume, intraoperative defibrillation requirement, levels of postoperative CTnT and CK-MB and postoperative LVEF. It did not increase CPB time, ACC time, postoperative CTnI, postoperative new-onset atrial fibrillation, postoperative stroke, postoperative IABP use rate, nor did it increase postoperative mechanical ventilation time, length of ICU stays, length of hospital stays, and mortality. Magnetic resonance imaging (MRI) likely represents the most thorough method to assess myocardial protection with special attention to the subendocardial myocardium and its viability. The recommendation that postoperative MRI scan be done from postoperative days 5 to 7 or another standardized time range to assess myocardial protection was proposed in future randomized trials of del Nido cardioplegia versus conventional cardioplegia [20].

Del Nido cardioplegia is a kind of single dose cardioplegia. The first infusion dose is generally 20 ml/kg and up to a maximum of 1 L, which can satisfy the cross-clamp time of 90 min. If the cardiac arrest time needs to be extended, the intraoperative infusion amount is generally added at the dose of 10 ml/kg. The results of several studies have shown that the perfusion volume of del Nido cardioplegia is significantly lower than that of blood-containing cardioplegia requiring repeated perfusion [21, 22]. In this review, 7 RCT studies involving intraoperative cardioplegia volume were systematically evaluated, and the results were consistent with published studies showing that the use of del Nido cardioplegia in adult cardiac surgery significantly reduced the use of cardioplegia. Less cardioplegia can reduce blood dilution in patients and reduces postoperative transfusion. The research objects in the ten RCT studies only included patients who underwent CABG surgery or valvular surgery. For complex operations with long operation time, such as operations on large vessels, there is no consensus on how del Nido cardioplegia should be used for additional perfusion after adult heart surgery exceeding 90 min and the interval of additional perfusion, which requires further study. A retrospective study found that the use of del Nido cardioplegia in adult cardiac surgery reduced the need for defibrillation after cardioversion following intraoperative circulatory arrest [23]. The results of this study support this conclusion. The success rate of spontaneous rhythm recovery after intraoperative circulation arrest in the del Nido cardioplegia group is higher than that in the cold blood cardioplegia group, and the need for defibrillation is lower.

Troponin is released from damaged cell membranes only after cardiac muscle cells have died, and both CTnT and CTnI are ideal for detecting myocardial damage [24]. CK-MB is an important biomarker in the diagnosis of myocardial injury, and it is used as a potential auxiliary detection index in combination with troponin to reflect the degree of myocardial injury [25]. Del Nido cardioplegia was associated with lower CTnT and CK-MB after cardiac surgery in a meta-study [26] that included only 2 RCT studies. A recent meta-analysis [27] about del Nido cardioplegia in myocardial protection included 3 RCT studies and showed no statistically significant difference in CTnT and CTnI levels 24 h after cardiac surgery between the DNC group and the CBC group. Among the 10 RCT studies included in this study, there were 3, 4 and 5 studies involving the levels of CTnI, CTnT and CK-MB at 24 h after surgery with complete data, respectively. The results showed that DNC significantly reduced the levels of CTnT and CK-MB compared with CBC. There was no significant difference in postoperative CTnI. Partial researches [21, 28] showed that del Nido cardioplegia were significantly better than the blood cardioplegia group in improving postoperative LVEF. Another retrospective study showed that del Nido cardioplegia were comparable to cold blood cardioplegia in patients with reduced ejection fraction (EF < 40%), with no difference in postoperative EF levels. This study systematically evaluates the postoperative LVEF levels of del Nido cardioplegia and cold blood cardioplegia, and the results supported that the postoperative ejection fraction of del Nido cardioplegia was comparable with cold blood cardioplegia. Further RCT researches are needed to investigate the protective effect of DNC on myocardium.

The base solution of Del Nido cardioplegia is a non-glucose solution, which can reduce perioperative blood glucose elevation and postoperative insulin use to a certain extent [29]. The optimization of blood glucose management indicates an improvement in clinical efficacy. In the 10 studies included, only 2 RCT study assessed intraoperative blood glucose levels. One study compared the intraoperative blood glucose levels at the 5th minute after removal of the aortic cross-clamp, the glucose values favor the del Nido cardioplegia, but statistical significance was not reached. Another study compared intraoperative baseline blood glucose and peak blood glucose levels respectively. Intravenous peak blood glucose levels and insulin requirements were significantly lower in the del Nido cardioplegia group.

Although del Nido cardioplegia has shown beneficial effects on myocardial protection in adult cardiac surgery, there was no significant difference between DNC and CBC in terms of clinical outcomes, including duration of mechanical ventilation, length of stays in ICU, length of hospital stays, and postoperative adverse events.

Limitations of this study: Inotrope requirements and intraoperative echocardiography were not assessed due to incomplete data [2]. Although all RCT studies were included in this study, some RCT assignment hiding and blinding methods were unclear, which may lead to bias in selection, implementation and measurement; [3] Among the studies included in this review, the volume of cardioplegia single dose, the interval and dose of additional infusion, and the mode of infusion could not be unified. Although sensitivity analysis was performed, heterogeneity still could not be eliminated; [4] The sample size of the included studies is small and the number of studies is small, so it is necessary to increase the sample size to obtain exact evidence.

## Conclusion

Existing evidence suggests that del Nido cardioplegia reduced volume of cardioplegia administration and attempts of defibrillation. The superior postoperative results in CTnT and CK-MB may provide a direction for further research on improvement of the composition of cardioplegia. Limited by the number and quality of included studies, the conclusions of this study need to be verified by multi-center randomized controlled studies with higher quality and large samples.

### Electronic supplementary material

Below is the link to the electronic supplementary material.

Supplementary Material 1

## Data Availability

All relevant data are within the manuscript and its Supporting Information files.

## Abbreviations

ACC: Aortic cross-clamp
AVR: Aortic valve replacement
CABG: Coronary artery bypass grafting
CBC: Cold blood cardioplegia
CI: Confidence interval
CK: MB-Creatinine Kinase-Myocardial Band
CPB: Cardiopulmonary bypass
CTn: Cardiac Troponin
DNC: Del Nido Cardioplegia
LVEF: Left ventricular ejection fraction
IABP: Intra-aortic balloon pump
ICU: Intensive care unit
MRI: Magnetic resonance imaging
RCT: Randomized clinical trial
RR: Relative risk
SD: Standard deviation
VS: Valve surgery
