# Supplementary material for: Del Nido cardioplegia versus cold blood cardioplegia in adult cardiac surgery: a meta-analysis of randomized clinical trials
Source: J Cardiothorac Surg. 2024 Jun 22;19:356. doi: 10.1186/s13019-024-02846-0 (PMC11193264; doi:10.1186/s13019-024-02846-0)
Supplement: Supplementary file 1 — Supplementary Material 1 [file 13019_2024_2846_MOESM1_ESM.docx]

Supplementary material

**Del Nido cardioplegia versus cold blood cardioplegia in Adult Cardiac Surgery: A meta-analysis of randomized clinical trials**

**Congcong Li et al.**

Supplementary Figures S1. Results of bias risk assessment for included studies.


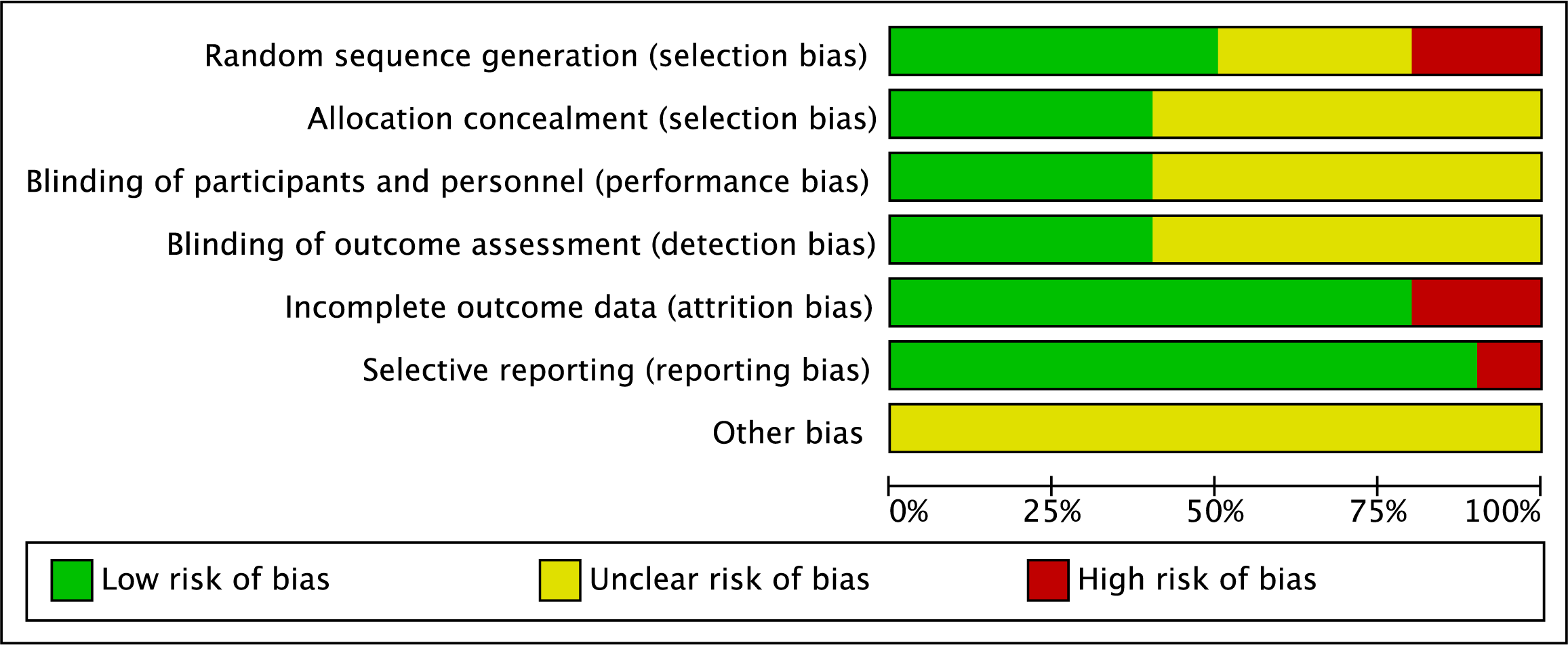


Supplementary Figures S2. Risk of bias summary for included studies.


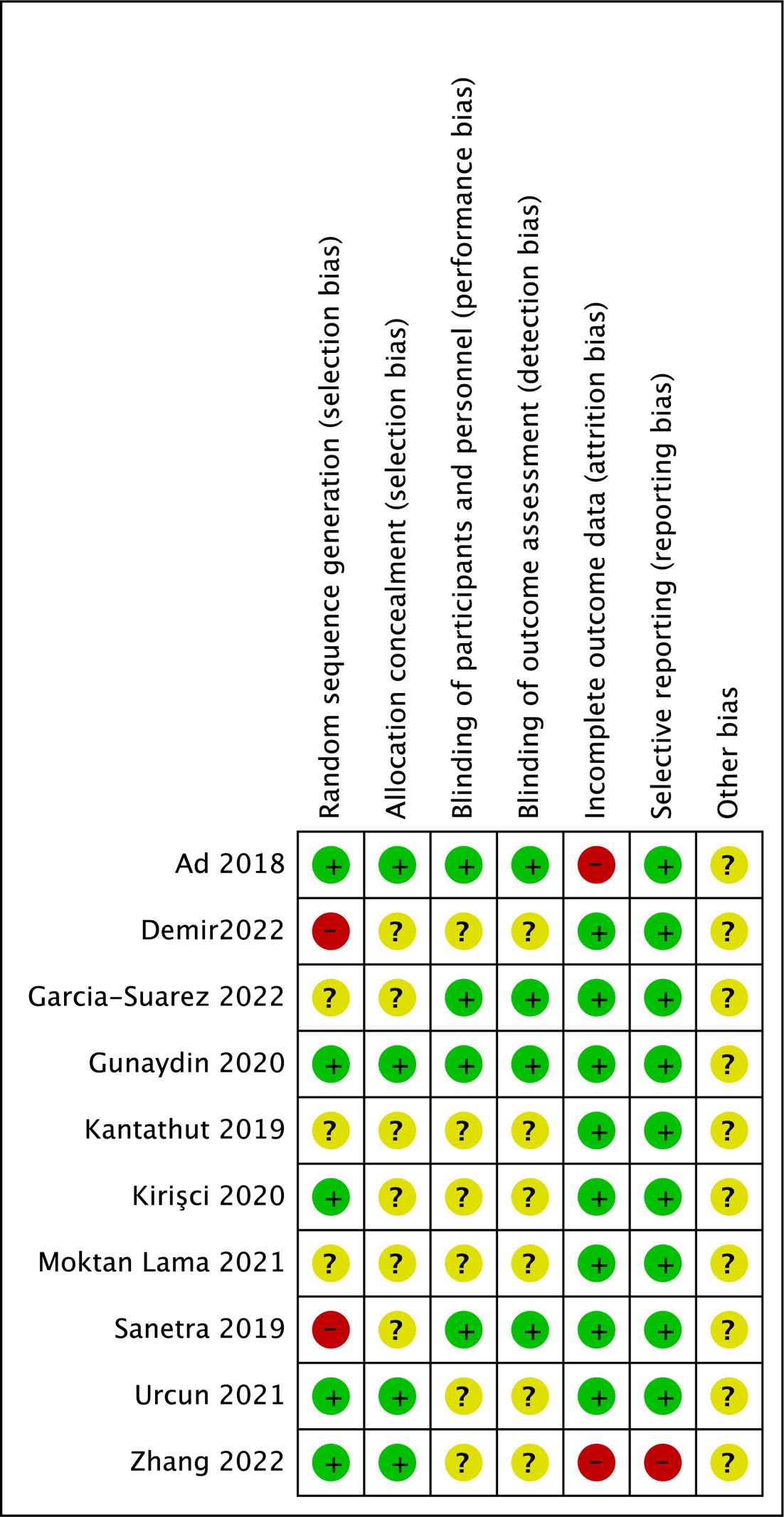


Supplementary Figures S3. Funnel plot. A: Volume of cardioplegia; B: Defibrillation after aortic cross-clamp removal; C: Postoperative Cardiac Troponin T (CTnT) at 24 hours after surgery; D: Postoperative Creatinine Kinase-Myocardial Band (CK-MB) at 24 hours after surgery.


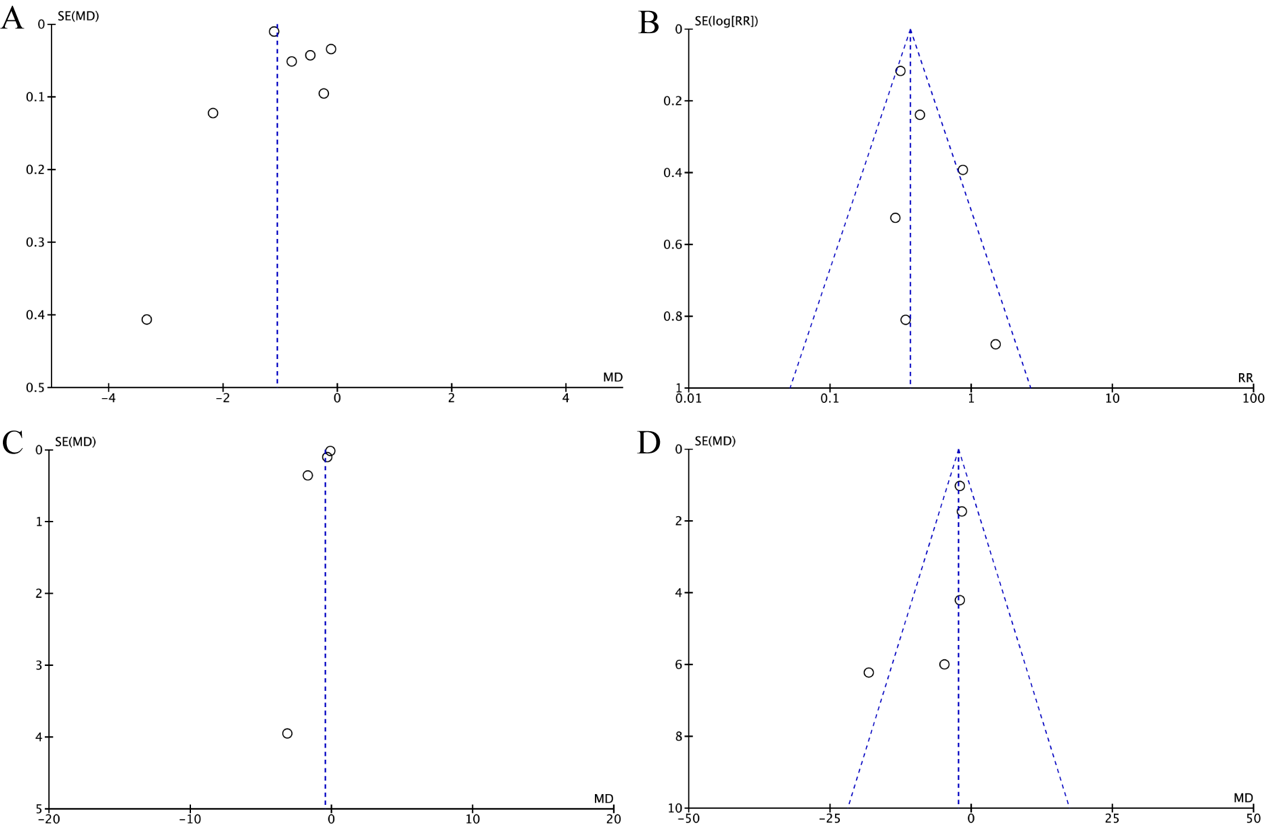


Supplementary Table S1. Summary of Cardioplegia Delivery.

| Cardioplegia | Del Nido Cardioplegia | | Cold Blood Cardioplegia | |
| --- | --- | --- | --- | --- |
|  | Cardioplegia Delivery | Temperature | Cardioplegia Delivery | Temperature |
| Ad 2018 | Initial dose: 1000mL  Subsequent dose:500mL (every 90 minutes or spontaneous return of electrical activity during the aortic cross-clamp period or left ventricular hypertrophy) | 6-10°C | Induction dose: 1000mL to 2000mL  Additional dose was given every 20 minutes. | 8-11°C |
| Sanetra 2019 | Initial dose: 20mL/kg (maximal dose of 1500 mL)  Subsequent dose: ¼ to ½ of initial dose  (the cross-clamp time exceeded 90 minutes) | 4°C | Initial dose: 15 mL/kg  Additional doses: 5 mL/kg (every 20 to 30 minutes or electrical activity was observed) | 4°C |
| Kantathut 2019 | Initial dosage: 20 mL/kg (maximum dose of 1000mL)  Subsequent dose: the surgeon decides (after 90 minutes of aortic cross-clamp time) | 2-8°C | Initial dose: 20 mL/kg  Additional doses: 10 mL/kg (every 20 minutes) | 4°C |
| Kirisci 2020 | Initial dosage: 1000mL  Subsequent dose was administered after 60 minutes. | 4°C | Initial dose: 1000mL  Additional doses: 500 mL (every 20 minutes) | 32°C |
| Gunaydin 2020 | Initial dosage: 20 mL/kg (myocardial arrest with aortic  cross-clamp time expected to be <90 min) | 4-8°C | Initial dose: 15 mL/kg  Additional doses: 15 mL/kg (every 25 minutes) | 4-8°C |
| Moktan Lama 2021 | Initial dosage: 20 mL/kg  subsequent dose: 10 mL/kg (every 90 minutes) | 4-6°C | Initial dosage: 20 mL/kg  Additional dose: 10 mL/kg (every 20 minutes) | 4-6°C |
| Urcun 2021 | Initial dosage: 1200mL  Subsequent dose: 600-800ml at 60^th^ minute (if aortic cross-clamp time exceeded 90minutes) | 4°C | Initial dosage: patient weight*10mL  Additional dose: 300mL (every 20-25 minutes)  Last dose: 300mL (before reperfusion) | / |
| Demir 2022 | Initial dosage: 1000mL  Subsequent dose: 500 ml at approximately the 60^th^ minute (ischemic duration was thought to exceed 90 minute) | 4-10°C | Initial dosage: 10-15mL/kg  Additional dose: 300mL (every 15-20 minutes) | 4-10°C |
| Garcia-Suarez 2022 | Initial dosage: 1000mL  Subsequent dose: 500 ml (every 90 minutes or spontaneous return of electrical activity during the aortic cross-clamp period) | 4°C | Initial dosage: 200mL  Additional dose: 100mL (every 20 minutes) | 4°C |
| Zhang 2022 | Initial dosage: 20mL/kg  Subsequent dose: 500 ml (every 90 minutes or necessary) | 4-6°C | Initial dosage: 20mL/kg  Additional dose: 10mL/kg (every 30 minutes) | 10-12°C |
